# Pulmonary infection caused by *Nocardia cyriacigeorgica* and influenza A virus coinfection in an immunocompetent patient: A case report

**DOI:** 10.1097/MD.0000000000051059

**Published:** 2026-10-23

**Authors:** Zhengfan Yu, Yanmei Zhang, Lijie Qiu, Leqiang Wang

**Affiliations:** a Weifang People’s Hospital, Shandong Second Medical University, Respiratory Medicine Center, Weifang, Shandong, China.

**Keywords:** *i*nfluenza A virus, *Nocardia cyriacigeorgica*, pulmonary infection, targeted next-generation sequencing

## Abstract

**Rationale:**

Pulmonary nocardiosis (PN) is an opportunistic infection caused by *Nocardia* species. It is characterized by a lack of specificity in both clinical symptoms and imaging findings. Most cases occur in individuals with compromised immune systems or underlying pulmonary diseases. In contrast, our patient did not present with any of these risk factors.

**Patient concerns:**

A 60-year-old immunocompetent male was admitted with a 5-month history of recurrent cough and expectoration. His respiratory symptoms worsened, and he developed a fever 9 days prior to hospitalization.

**Diagnoses:**

*Nocardia cyriacigeorgica* was identified in the sputum culture, as well as in the targeted next-generation sequencing and culture of the bronchoalveolar lavage fluid, while influenza A virus was detected by nucleic acid detection.

**Interventions:**

Along with antiviral therapy with peramivir, a combination antibiotic regimen of trimethoprim-sulfamethoxazole (TMP-SMX), etimicin, and meropenem was administered during hospitalization. Following discharge, oral TMP-SMX was continued as maintenance therapy.

**Outcomes:**

Regular follow-ups with the patient revealed that subsequent chest computed tomography (CT) scans showed a gradual reduction in the extent of the lesions, and the patient demonstrated a good clinical response and radiological improvement.

**Lessons:**

This case highlights the diagnostic complexity of PN. The radiological manifestations are diverse, commonly including pulmonary consolidation, nodules, and cavities, making it difficult to differentiate from other diseases. Early, precise diagnosis and prompt combined therapy are of vital importance in severe *Nocardia* pulmonary infection.

## 1. Introduction

*Nocardia* species are opportunistic pathogens primarily, affecting individuals with compromised cell-mediated immunity or other immunosuppressive conditions.^[1]^ The genus *Nocardia* comprises over 100 validly published species, with more than 50 recognized as pathogenic to human. Common clinical pathogen include members of the *Nocardia asteroides* complex (e.g., *N asteroides* itself), *Nocardia brasiliensis*, and *Nocardia otitidiscaviarum.*^[2]^ *Nocardia* primarily invades the host via the respiratory tract, with clinical manifestations often presenting as pulmonary nocardiosis (PN). Infection with *Nocardia cyriacigeorgica*, a relatively rare pathogen, is predominantly associated with key risk factors including solid organ transplantation, prolonged corticosteroid use, and the presence of underlying chronic pulmonary diseases.^[3,4]^ We present a case of PN in an immunocompetent individual without underlying chronic respiratory conditions, and review the relevant literature to enhance the understanding of its clinical management.

## 2. Case report

This case report was approved by the Institutional Review Board of the First Affiliated Hospital of Shandong Second Medical University. A 60-year-old male farmer presented with a 5-month history of productive cough and recurrent expectoration, accompanied by right-sided chest pain and fever. A chest computed tomography (CT) scan performed at a local county hospital revealed cloud-like and patchy opacities in both lungs. The patient initially received empirical antibiotic therapy for 1 week, resulting in normalization of his body temperature and alleviation of the cough. However, 9 days prior to admission, he experienced a recurrence of fever (*T*-max 39.0°C) accompanied by chills. Seven days later, he developed a cough with yellow-white mucoid sputum, nasal congestion, occasional rhinorrhea, and chest tightness with dyspnea, which worsened upon exertion but improved at rest. He also reported right-sided non-radiating chest pain and transient nausea without vomiting. Despite receiving treatment with moxifloxacin and cefoperazone-sulbactam at a local hospital, his symptoms showed no significant improvement. On January 29, 2025, he was admitted to our hospital with a diagnosis of “pulmonary infection, suspected lung cancer.” The patient had no history of smoking, illicit drug use, or known immunocompromised conditions. He denied previous COVID-19 infection, tuberculosis, corticosteroid use, diabetes, or surgical trauma. There was no family history of similar respiratory symptoms.

Physical examination revealed a body temperature of 38.5°C, pulse rate of 88 beats/min, respiratory rate of 25 breaths/min, and blood pressure of 130/82 mm Hg. Assessment of the respiratory system indicated mild tachypnea with bilaterally diminished breath sounds. Auscultation detected scattered, coarse crackles in both lung fields. Laboratory tests showed an elevated white blood cell count of 28.13 × 10^9^/L with a neutrophilic predominance (93.7%). Platelet count was within normal limits at 305 × 10^9^/L, whereas the erythrocyte sedimentation rate (ESR) was significantly elevated to 96.00 mm/h. Fibrinogen levels were increased to 7.13 g/L, and prothrombin time was prolonged to 17.9 seconds. Additionally, procalcitonin was elevated to 4.49 ng/mL, and C-reactive protein (CRP) exceeded 150 mg/L. A multiplex nucleic acid test for 13 respiratory pathogens was positive for influenza A virus. Chest CT scan demonstrated bilateral pulmonary infiltrates consistent with inflammatory changes (Fig. 1A–C). All infection-related screenings (including Mycobacterium tuberculosis DNA, HIV, hepatitis B, syphilis, and hepatitis C) returned negative results. Serum immunoglobulin (IgG, IgA, IgM) levels all fell within the normal reference range, and tumor marker tests (carcinoembryonic antigen, cytokeratin 19 fragment, neuron-specific enolase, and squamous cell carcinoma-associated antigen) were also negative, indicating no definite abnormalities within the scope of the above screenings.

**Figure 1.**
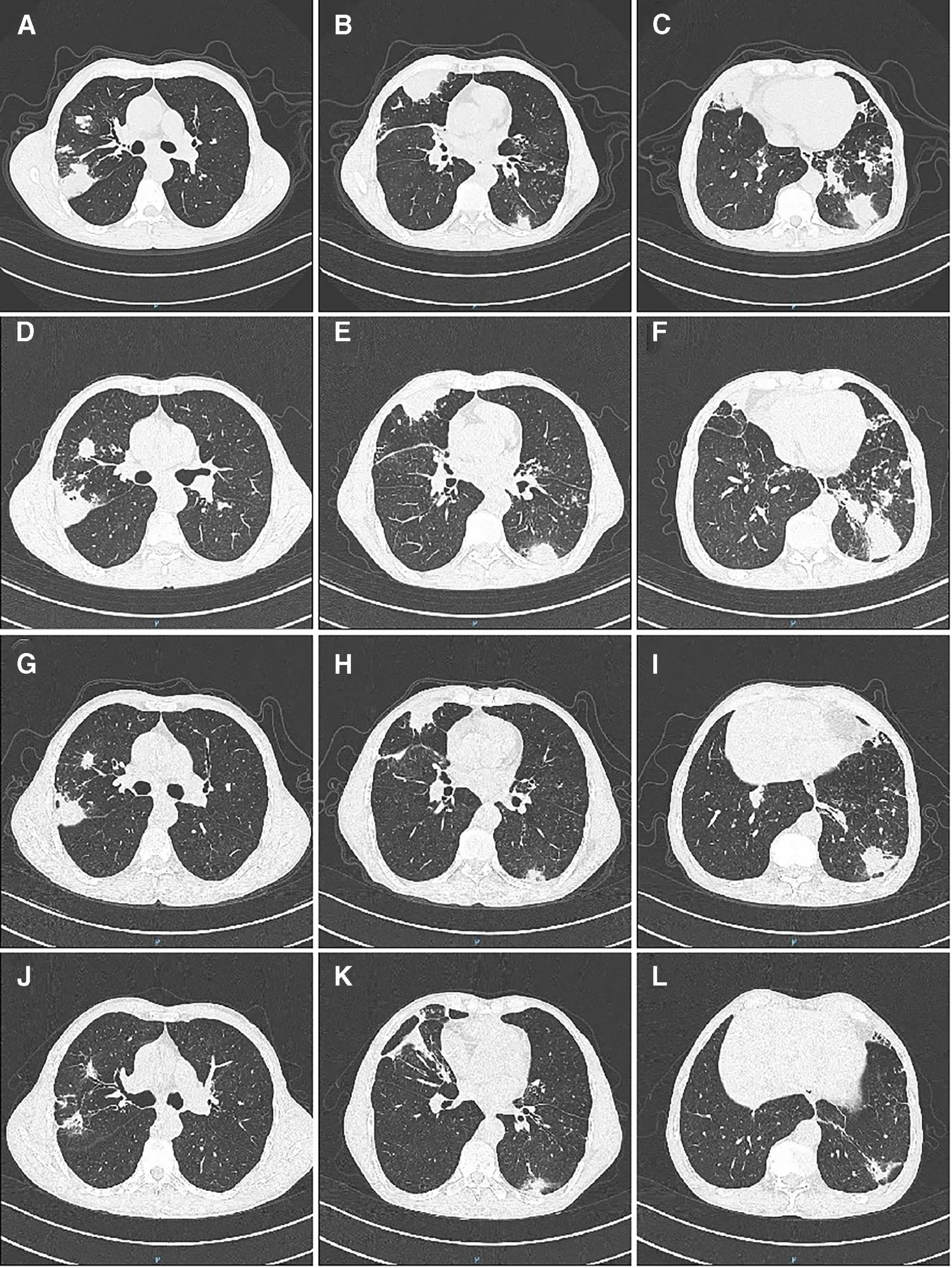
(A–C) Chest CT findings on the day of admission. (D–F) On day 5 of TMP-SMX treatment. (G–I) On day 23 of TMP-SMX treatment. (J–L) On day 62 of TMP-SMX treatment. CT = computed tomography, TMP-SMX = trimethoprim-sulfamethoxazole.

On hospital day 1, empirical antimicrobial therapy with ertapenem (1 g once daily intravenously) was initiated for the management of suspected severe community-acquired pneumonia. Concurrently, given the patient’s positive test for influenza A virus, antiviral therapy with peramivir (0.3 g once daily by intravenous infusion) was administered along with other symptomatic treatments. Despite this combined anti-infective and antiviral regimen, the patient remained febrile, with a maximum temperature exceeding 39°C. To further characterize the pulmonary abnormalities, a contrast-enhanced chest CT scan was performed, revealing multiple patchy and nodular opacities in both lungs. These findings raised suspicion of underlying malignancy complicated by superimposed infection (Fig. 2). Additional observations included bilateral bronchiectasis with signs of infection and the presence of mucus plugs in the airways. A blood culture obtained on February 4, 2025, showed no microbial growth after 5 days of incubation. To directly assess the airway and obtain diagnostic samples, a bronchoscopy was performed on hospital day 6. The bronchoscopic examination revealed a moderate amount of white purulent secretions within the bronchi of both lungs; however, no endobronchial masses or active bleeding sites were identified (Fig. 3). A bronchoalveolar lavage fluid (BALF) sample was collected and analyzed by targeted next-generation sequencing (tNGS), which detected *N cyriacigeorgica*. This critical finding was immediately communicated to the microbiology laboratory, prompting an extension of the conventional bacterial culture incubation period to facilitate the growth of this fastidious organism. Following 5 days of antiviral treatment, a repeat test for influenza A virus returned negative.

**Figure 2.**
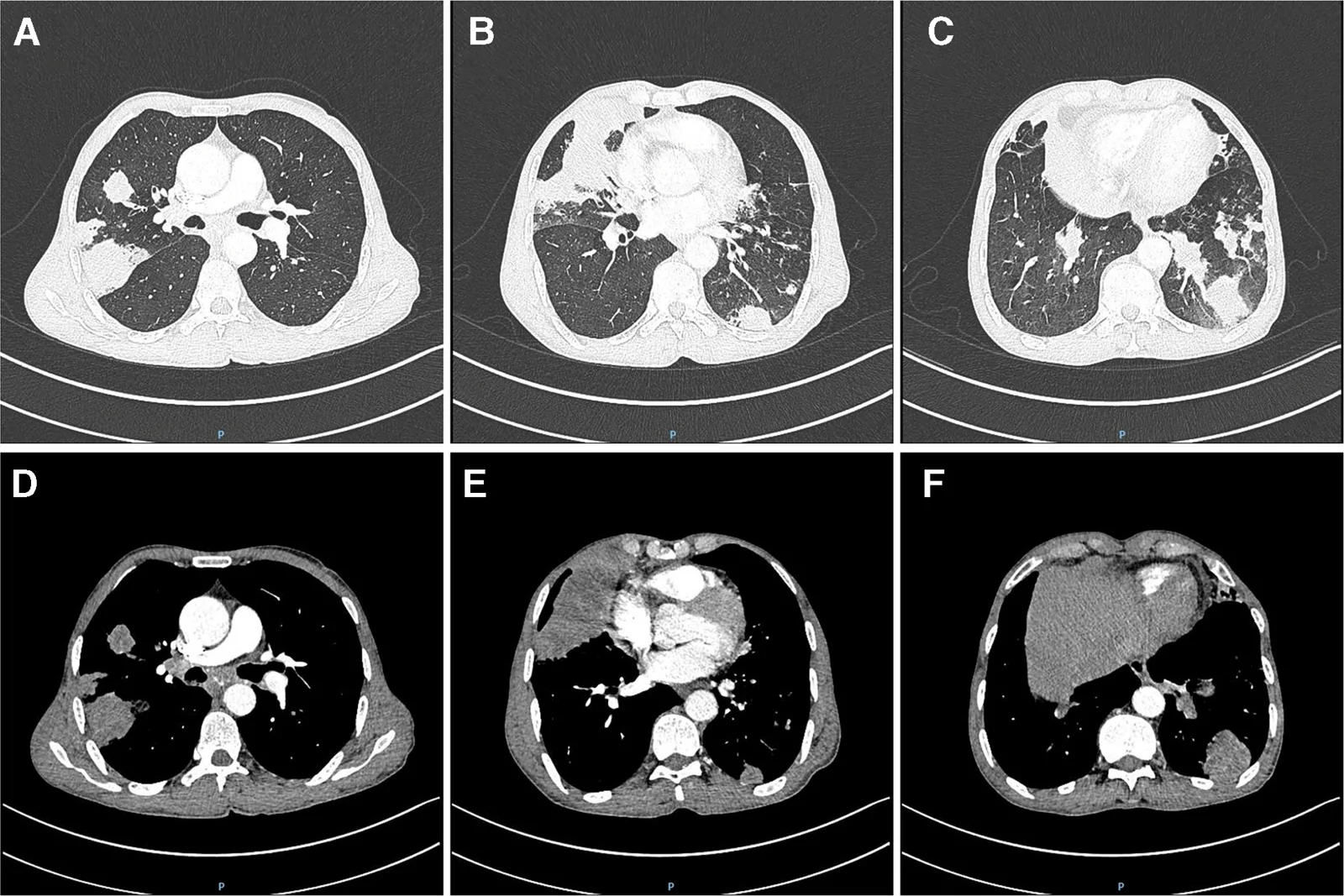
Multiple patchy and nodular opacities in both lungs, considering co-existing tumor and inflammation.

**Figure 3.**
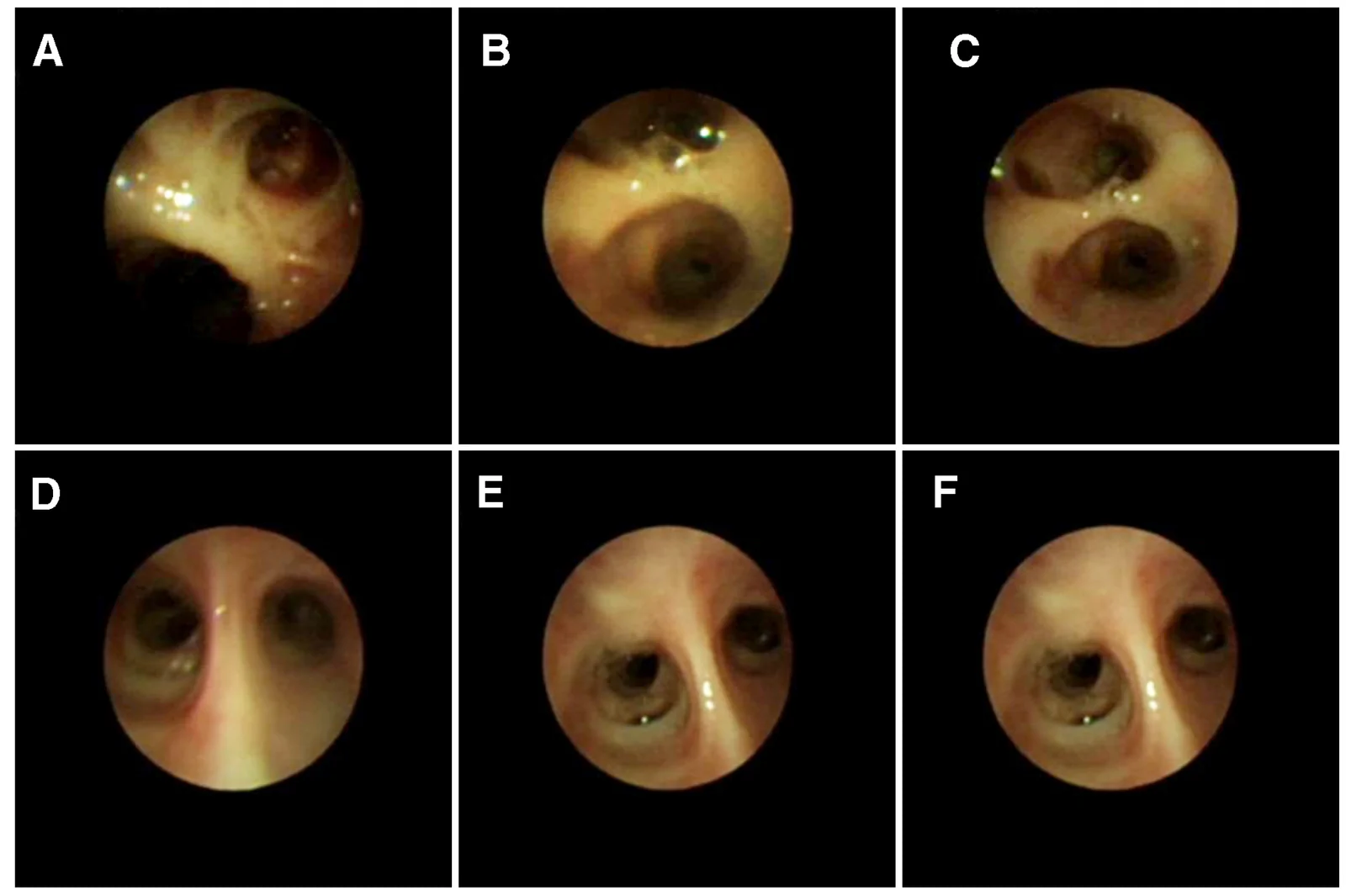
The trachea is patent with slight mucosal congestion. A moderate amount of white purulent secretions is observed in the bronchi of both lungs, with no bleeding or neoplasm identified. A small amount of secretions is also noted.

On hospital day 7, following the tNGS report indicating *N cyriacigeorgica,* the antimicrobial regimen was adjusted to a triple-antibiotic therapy targeting nocardial infection. The patient received oral trimethoprim-sulfamethoxazole (TMP-SMX) at a dosage of 2 double-strength tablets (each containing 160 mg trimethoprim and 800 mg sulfamethoxazole) 4 times daily, combined with intravenous etimicin (300 mg once daily) and meropenem (1.0 g every 8 hours). To promote the growth of Nocardia, Sabouraud dextrose agar supplemented with hemin and NAD was used, and the culture was incubated at 35°C under microaerophilic conditions for 7 days. On February 5, 2025 (hospital day 7), sputum culture (semi-quantitative result: ++) revealed growth of *Nocardia*. On hospital day 8, bronchoalveolar lavage fluid quantitative culture yielded a colony count of > 10^4^ CFU/mL, identified as *N cyriacigeorgica* by MALDI-TOF MS (Nocardia was also observed on sputum smear). Susceptibility testing performed according to CLSI M24 standards using the broth microdilution method showed that the isolate was sensitive to TMP-SMX. To further characterize the pulmonary opacities and exclude underlying malignancy, a CT-guided percutaneous lung biopsy was performed on February 7, 2025. Histopathological examination of the biopsy specimen revealed acute and chronic inflammation with microabscess formation and granulomas, accompanied by reactive alveolar epithelial hyperplasia (Fig. 4). No malignant cells were identified. Analysis of lymphocyte subsets on February 8 showed a decreased total T lymphocyte count (absolute count 453/μL). A subsequent 7-day fungal culture returned no growth. After 5 days of the targeted anti-nocardial therapy, the patient was afebrile. Meanwhile, the chest CT scan demonstrated a reduction in the size of the lesion compared to the previous study (Fig. 1D–F). By hospital day 14, he had become afebrile, with a general improvement in symptoms, decreased moist rales on lung auscultation, and significantly reduced sputum production. The patient was discharged on day 14 at his request to continue oral TMP-SMX. At the 4-week outpatient follow-up, chest CT demonstrated mild regression of pulmonary infiltrates (Fig. 1G–I). Physical examination revealed clear breath sounds bilaterally, with no audible rales. The patient was advised to continue TMP-SMX. A follow-up chest CT 2 months after discharge showed significant resolution of the pulmonary lesions (Fig. 1J–L). T-cell count normalized to 980/µL, with complete blood count and CRP also within normal ranges. The treatment was deemed highly effective, and continuation of oral TMP-SMX was recommended.

**Figure 4.**
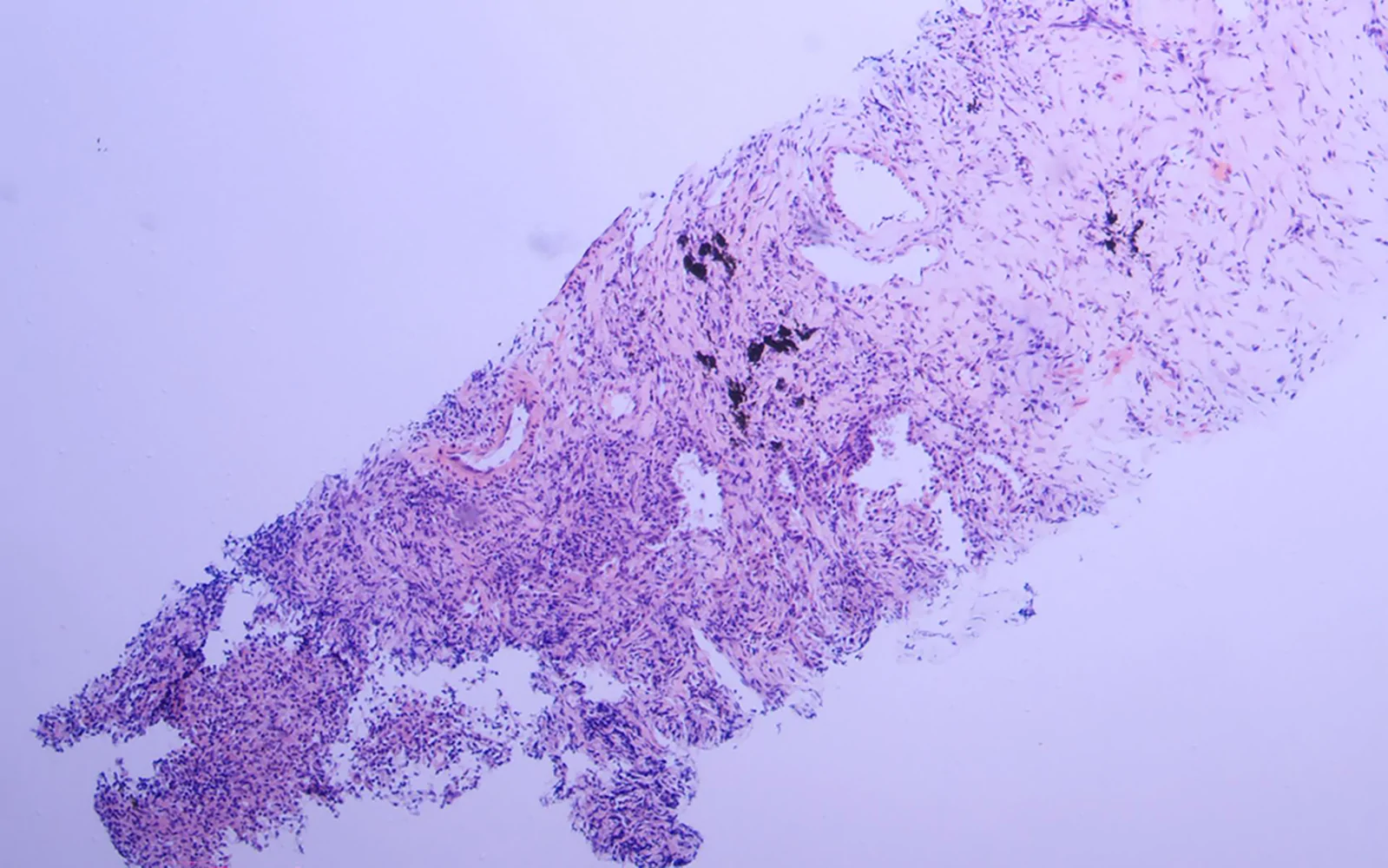
(Right lung mass puncture) Lung tissue with acute and chronic inflammation, accompanied by microabscess formation, local fibrous tissue hyperplasia, and focal carbon deposition. Granuloma formation is observed, with hyperplasia of surrounding alveolar epithelium (×100).

## 3. Discussion

*Nocardia* is a genus of actinomycetes, which are Gram-positive, obligate aerobic, and exhibit weak acid-fastness, with a broad natural habitat that primarily includes soil and decomposing plant material. *N cyriacigeorgica* was first isolated from a patient with chronic bronchitis in 2001. It typically causes infections in immunocompromised and debilitated patients and is considered an opportunistic pathogen.^[5]^ However, approximately one-third of *Nocardia* infections may also occur in immunocompetent individuals.^[1]^ In this patient, the decreased T-cell count (453/µL) on admission was considered a temporary decline due to acute infection and stress, as it promptly returned to normal (980/µL) after infection control, and the patient had no clinical manifestations or history of underlying chronic immunodeficiency. This further supports that nocardiosis can also occur in hosts with essentially intact immune status.

Etiological diagnosis constitutes the cornerstone for confirming PN. Traditional pathogenic detection primarily relies on the isolation and culture of specimens such as sputum, BALF, or biopsied lung tissue.^[6]^ Following bacterial culture, identification by mass spectrometry is considered the “gold standard” for differentiating *Nocardia* subspecies.^[7]^ While sputum cultures offer the advantages of noninvasiveness and low cost for initial pathogen screening, their diagnostic utility can be limited by potential contamination from the upper airways, leading to variable accuracy. BALF offers several advantages, including relative procedural simplicity, a low risk of contamination, and reasonably reliable results. Consequently, BALF is particularly suitable for etiological diagnosis in immunocompromised patients, cases of initial treatment failure, suspected infections with specific pathogens (such as *Mycobacterium tuberculosis*, fungi, and *Pneumocystis jirovecii*), and the differential diagnosis of noninfectious diffuse lung diseases. In contrast, percutaneous lung biopsy carries risks of complications such as pneumothorax or hemorrhage. To enhance the diagnostic yield, a combined approach integrating sputum culture with BALF analysis was implemented.

During the diagnostic workup of this case, we systematically considered a range of differential diagnoses, including bacterial pneumonia, pulmonary tuberculosis, fungal infections (such as aspergillosis), malignancy, and organizing pneumonia. However, subsequent investigations ruled out these possibilities: tuberculosis was excluded by a negative TB-DNA test and the absence of relevant exposure or medical history; malignancy was deemed unlikely as lung biopsy revealed no malignant cells, and relevant tumor markers were within normal ranges; fungal infection was not supported by negative fungal cultures and a lack of typical clinical features suggestive of fungal disease; rheumatologic or autoimmune etiologies were considered unlikely given normal inflammatory and autoimmune markers (e.g., ANA, RF, and anti-CCP). Although imaging revealed a mass-like lesion, the findings lacked characteristic histopathological or radiographic features of organizing pneumonia. Ultimately, the diagnosis of *N cyriacigeorgica* infection was confirmed through targeted NGS of BALF and sputum culture.

*Nocardia* species exhibit slow growth rates; even when present in substantial quantities in specimens, colony formation typically requires 3 to 5 days of culture. For specimens with low bacterial loads, an extended incubation period – sometimes exceeding 3 weeks – is necessary to observe visible growth.^[8]^ The frequent false-negative results of conventional culture methods delay diagnosis and targeted therapy, with implications including increased disease transmission, morbidity, and mortality.^[9,10]^ With advances in molecular technologies, tNGS and other molecular diagnostic methods are being increasingly applied to the etiological diagnosis of pulmonary infections. Compared with conventional methods that rely on isolate culture and mNGS, which nonspecifically sequences all nucleic acids, tNGS – by targeting predefined pathogen-specific markers – offers advantages in high sensitivity, efficiency, and relatively lower cost.^[11,12]^ In the present case, tNGS provided critical clues for the early identification of Nocardia infection, a result that was subsequently confirmed by conventional sputum culture.

PN typically follows a subacute or chronic course, manifesting with nonspecific symptoms such as fever, cough, chest pain, night sweats, and weight loss. Common thoracic CT findings include consolidation, nodules, mass-like opacities, and pleural effusion. Notably, mediastinal and hilar lymphadenopathy is an atypical feature of this infection.^[13]^ The combination of these nonspecific clinical and radiological features often leads to misdiagnosis as common pulmonary infections, tuberculosis, lung abscess, or malignancy.^[14-16]^ In the present case, the presence of multiple irregular masses posed a significant diagnostic challenge, as the radiographic appearance was difficult to distinguish from pulmonary aspergillosis or lung cancer. A CT-guided percutaneous core needle biopsy was therefore performed to exclude malignancy. The definitive diagnosis was ultimately established by concurrent testing of BALF using both tNGS and conventional culture, which guided subsequent targeted antimicrobial therapy. The patient’s significant clinical improvement and remarkable radiologic resolution on follow-up further corroborated the diagnostic findings.

The management of nocardiosis is guided by the infection site, host immune status, and antimicrobial susceptibility profiles. Sulfonamides, particularly TMP-SMX, remain the first-line therapeutic option for most *Nocardia* infections. These agents inhibit bacterial folate metabolism, thereby disrupting nucleic acid synthesis.^[17]^ In cases of intolerance, resistance, or severe disease, alternative agents such as amikacin, imipenem, and linezolid are recommended; among these, carbapenems and linezolid demonstrate broad-spectrum activity against most pathogenic *Nocardia* species.^[18,19]^ For immunocompromised hosts or those with disseminated disease, combination therapy is often employed based on guidelines and clinical studies to enhance coverage and improve outcomes. The duration of antimicrobial therapy typically exceeds 6 months and is influenced by the infection severity, immune status, and clinical response. Central nervous system involvement usually mandates a more prolonged course, frequently extending to 12 months or longer.^[1,20]^ In the present case, the administration of TMP-SMX led to the resolution of fever and clinical symptoms, a response consistent with the antimicrobial susceptibility profile and supporting the treatment approach. The patient’s clinical course, diagnostic workup, and therapeutic interventions throughout the hospitalization are summarized in Figure 5.

**Figure 5.**
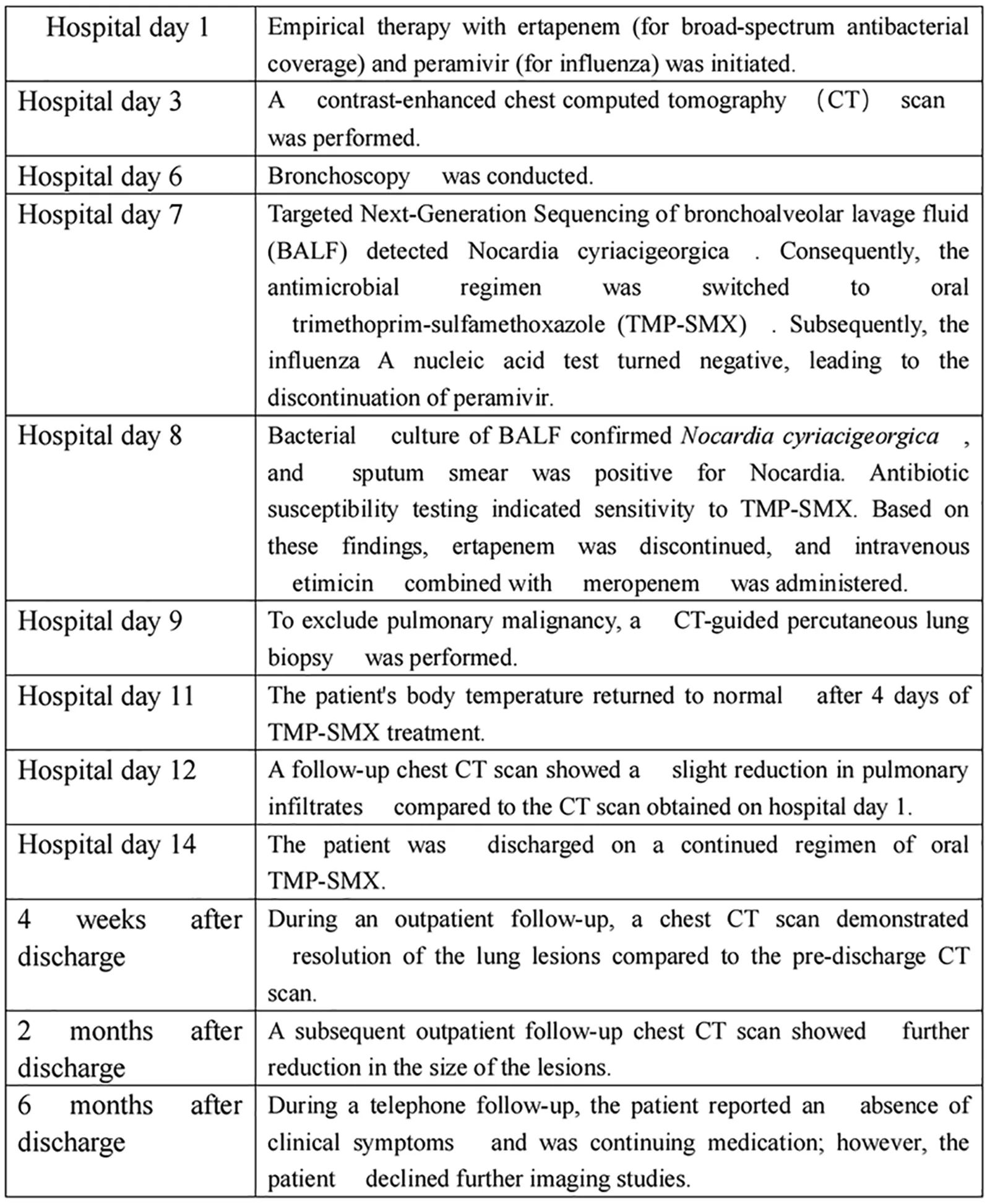
Diagnosis and intervention chart. BALF = bronchoalveolar lavage fluid, CT = computed tomography, TMP-SMX = trimethoprim-sulfamethoxazole.

In conclusion, PN is an infectious disease that warrants heightened clinical awareness, as its nonspecific symptoms and imaging findings frequently result in misdiagnosis. Consequently, the incorporation of advanced molecular diagnostics, such as tNGS, is critical for achieving a rapid and accurate etiological diagnosis. TMP-SMX is the cornerstone of treatment; however, effective management must be individualized according to susceptibility profiles, infection location, and the patient’s immune status. Ultimately, successful outcomes rely on an individualized therapeutic strategy and meticulous clinical monitoring to guide subsequent adjustments. Therefore, for chronic pneumonia that presents with a clinical–radiological mismatch (e.g., nodules or cavitation on imaging) or fails to respond to empirical antimicrobial therapy, clinicians should consider the possibility of Nocardia infection and proactively request the microbiology laboratory to extend culture incubation and/or perform molecular testing.

## Author contributions

**Conceptualization:** Lijie Qiu.

**Data curation:** Yanmei Zhang.

**Investigation:** Zhengfan Yu, Yanmei Zhang.

**Supervision:** Leqiang Wang.

**Writing – original draft:** Zhengfan Yu.

**Writing – review & editing:** Zhengfan Yu, Lijie Qiu, Leqiang Wang.
